# Investigating the causes of patient anxiety at induction of anaesthesia: A mixed methods study

**DOI:** 10.1177/1750458920936933

**Published:** 2020-07-08

**Authors:** Andrew B Lumb, Gary J Latchford, Hilary L Bekker, Anna R Hetmanski, Caroline R Thomas, Claire E Schofield

**Affiliations:** 1 Leeds Institute of Biological and Clinical Sciences, Leeds, UK; 2 Leeds Institute of Health Sciences, Leeds, UK; 3 Department of Psychological and Social Medicine, University of Leeds, Leeds, UK; 4 Airedale NHS Foundation Trust, Keighley, UK; 5 St James’s University Hospital, Leeds, UK

**Keywords:** General anaesthesia / Anxiety / Physiological stress / Preoperative care / Healthcare team

## Abstract

**Aim:**

To investigate patient anxiety at anaesthetic induction and whether this is affected by anaesthetic room interventions.

**Methods:**

A mixed methods study was carried out: pre-induction interventions were directly observed. Patient anxiety was assessed quantitatively with cardiovascular changes, the visual analogue scale and the state-trait anxiety inventory. Interviews allowed qualitative assessment.

**Results:**

Patient-reported anxiety did not correlate with cardiovascular changes. Anaesthetic room interventions were not predictive of anxiety. Postoperative interviews identified five sources of anxiety, mostly related to preparation for surgery. Staff responses to anxiety were also highlighted.

**Discussion:**

Patient-reported anxiety and its biological response are not correlated. Pre-induction interventions do not contribute to anxiety. Anxiety levels at induction are similar to or lower than earlier in the preoperative period.

**Conclusions:**

On induction of anaesthesia, patients have little control over their situation but are actively reassured and distracted by theatre staff. Our data suggest staff are good at this. More could still be done to reduce preoperative sources of anxiety.

**Provenance and Peer review:** Unsolicited contribution; Peer reviewed; Accepted for publication 3 June 2020.

## Introduction

Anxiety is an uncomfortable feeling of nervousness or worry about something that is happening or might happen in the future and is common in the perioperative period (Wetsch et al 2009). It is a preventable risk factor for postoperative complications (Stamenkovic et al 2018) and has been a recognised entity for over 40 years. Causes of perioperative anxiety may be considered in broad categories: the fear of the unknown, the fear of being ill or in pain in the recovery period and the fear of dying. Specific patient concerns include fear about provision for their families or about the type of surgery or anaesthesia to be performed and the potential loss of independence (Caumo et al 2001). A fear of waking under anaesthesia, experiencing injections, application of a facemask and in the case of regional anaesthesia, seeing the body being cut open have also been identified as specific sources of anxiety (Mitchell 2008). While some anxiety may be considered a normal response to a stressful event, it may also be an abnormal reaction, akin to an irrational fear. High levels of anxiety are recognised as occurring in response to interventions and are shown to impair recovery and contribute to lack of adherence to treatment regimens (Bekker et al 2003). Greater perioperative anxiety is also associated with an increased incidence of nausea and vomiting, higher postoperative pain scores, an increased length of hospital stay (Celik & Edipoglu 2018) and possibly postoperative delirium (Van Grootven et al 2016). Factors associated with increased preoperative anxiety levels can be considered as sociodemographic factors, psychosocial factors and the type of surgery or anaesthesia. Disease-or operation-specific studies have variably found associations between many factors and anxiety, including smoking status, educational level, diagnosis and time elapsed from diagnosis to surgery; those most consistently associated with a greater incidence and degree of anxiety are younger age, female gender, lack of sleep, first time surgery, a previous history of cancer and those undergoing gynaecology and aesthetic surgery (Caumo et al 2001, Erkilic et al 2017). Ameliorating factors include a history of prior surgery, a good social support structure and carrying out the anaesthetic assessment in an outpatient setting. The degree of preoperative anxiety may also impact an individual’s coping behaviour and indirectly affect postoperative outcome as a result. Information giving and educational interventions have been instigated to lessen anxiety and improve patient experience although the timing and format of this information does not affect perioperative anxiety (Hounsome et al 2017). Pharmacological measures such as benzodiazepines or pregabalin, psychological preparation including cognitive behavioural therapy (Powell et al 2016) and non-pharmacological methods such as music therapy (Hole et al 2015) have also been shown to be beneficial. Evaluating preoperative anxiety allows better tailoring of analgesia in the postoperative period and a better patient experience (Ali et al 2014). Validated measures of patient anxiety are well established in clinical practice with several validated questionnaires available including the State-Trait Anxiety Inventory (STAI) (Spielberger et al 1983), Hospital Anxiety and Depression Scale and the Visual Analogue Scale for Anxiety (VASA) (Hornblow & Kidson 1976). The scale of the problem may be underappreciated as anxiety can be present in up to 85% of day-case patients and in a recent study of over 15,000 patients (Walker et al 2016), anxiety was most frequently cited (by 33%) as the worst aspect of the perioperative experience.

## Background

National guidance aiming to make anaesthesia safer has led to an increase in the technology incorporated into everyday practice. These standards have altered the way we interact with our patients but many may be considered intrusive, for example the alarms of our monitoring equipment (‘audible alarms must be enabled before anaesthesia commences’, Checketts et al 2016: p86). The use of facemask preoxygenation has also been adopted into routine preoperative practice. These mandatory safety measures may inadvertently increase the anxiety levels of our patients and reduce the opportunities we have to address patient concerns.

The purpose of this study was to investigate factors associated with patient anxiety at the moment general anaesthesia is induced. Many studies, detailed in the ‘Introduction’ and ‘Discussion’ sections, have studied preoperative anxiety up to an hour before surgery, but few at the point when the patient loses consciousness. We aimed to quantify anxiety at this moment, and investigate the impact on patient anxiety of various safety procedures and patient–staff interactions.

## Methods

Research Ethics Committee approval was granted prior to starting the study (REC 16/LO/1936 IRAS No. 210363) and all patients provided written consent. We used a mixed methods observational cohort study design including:

- Observational methods to record clinical interventions immediately before induction of general anaesthesia.
- Questionnaire methods to assess patient-reported anxiety and experience of care.
- A semi-structured interview to provide a more in-depth understanding of anxiety, perception of causes and possible impact of pre-induction interventions.

### Participants

The study was undertaken in 2017 at a single urban teaching hospital where surgical services include colorectal, urology, thoracic, hepatobiliary, gynaecological and upper gastrointestinal specialities. The hospital is a regional oncology centre so a majority of procedures are for cancer management. All elective patients attend a preassessment clinic several days prior to surgery where they receive written information about their general anaesthetic (GA) and surgery where appropriate. Most undergo day-of-surgery admission to a designated admissions lounge area, from where they go directly to theatre. Two of the 12 theatres used for major elective surgery do not have anaesthetic rooms (ARs).

Written consent was taken on the morning of surgery. Participants were adult patients undergoing a GA for inpatient elective surgery. Exclusions were patients lacking capacity or the ability to complete the consent form and/or questionnaire and interview methods; patients taking drugs affecting heart rate and blood pressure; patients receiving neuraxial block or lines sited awake before induction of anaesthesia and patients requiring ongoing intravenous opioids or sedative drugs postoperatively, eg morphine via a patient-controlled device.

### Sample size

Castro et al (2010) suggest there is no definitive guidance on sample size estimates for surveys employing mixed methods; qualitative methodologies suggest that between six and 20 participants and quantitative methodologies suggest between 40 and 400 participants meet the needs of different types of analyses. We estimated 40 participants for the observation-questionnaire component of the study and ten participants for the qualitative component, to be sufficient to address our research objectives. The participants selected for interview were chosen to be representative of our overall study cohort for age, sex and previous anaesthetic experience, achieved by monitoring these factors during the recruitment period and selecting patients with the required factors for recruitment or interview. These three factors were identified after discussion within the research team and consultation with three clinical colleagues as the most likely to affect anxiety levels in patients. The same investigator carried out these interviews with participants prior to discharge from the hospital. Interviews lasted up to 20min.

### Data collection tools

Three study tools were developed and piloted for the study to elicit systematically the different types of data: a Clinical Observation Checklist was used alongside patient notes to record patient demographic details and clinical indicators at induction of anaesthesia (see supplemental Appendix 1); the Patient Reported Experience of Preoperative Anxiety Questionnaire (see supplemental Appendix 2) was used to elicit patients’ recall of their preoperative anxiety and care; the Patient Reported Experience of Preoperative Anxiety Interview Schedule (see supplemental Appendix 3) was used to elicit patient views and experiences of preoperative anxiety.

The following variables were recorded:

1. Patient characteristics: age, sex and a whether or not they had had a previous GA, to allow selection of participants for structured interviews to ensure these were representative of the whole cohort.
2. Pre-induction interventions: induction of GA in an AR or operating theatre (OT); the number of attempts at cannulation before induction classified into one or more than one; preoxygenation performed classified as none, via a loose-fitting facemask or a tight-fitting facemask; audible alarm activity classified into 0 – no alarm noise throughout, 1 – occasional background/quiet alarm, 2 – occasional loud alarm, 3 – intermittent but repeated and intrusive alarms, 4 – constant loud alarms with no significant quiet periods and the staff responses to the alarms, ie their interactions with the patient in response to any audible alarms, classified into 0 – alarms silenced and the patient reassured, 1 – alarms silenced but not commented upon to patient or 2 – alarms ignored by all staff.
3. Patient clinical responses: heart rate and blood pressure in the preassessment clinic and maximum values achieved while awake prior to induction, recorded as the product of both (rate–pressure product (RPP)).
4. Anxiety in post-anaesthesia care unit (PACU): short-form anxiety inventory of six items including calm, tense, upset, relaxed, content and worried rated on a four point scale (Marteau & Bekker 1992); VASA for anxiety (zero-maximum anxiety); free text response to prompts about being worried, relaxed and role of the clinical setting in ameliorating or enhancing anxiety (see supplemental Appendix 2).

### Procedure

Participants were selected by convenience sampling. An investigator was present during induction of anaesthesia to observe the preoperative interventions and clinical responses and in the PACU to assess patients’ anxiety as described above.

Ten participants completed the Patient Reported Experience of Preoperative Anxiety Interview Schedule (see supplemental Appendix 3) carried out by a single investigator on the first postoperative day. Interviews were recorded and then transcribed by the same investigator for analysis.

### Analysis

Quantitative data were analysed using SPSS Version 23.0.0.2. Pearson’s correlation coefficient was used to compare the three measures of anxiety (STAI, VASA and RPP). An independent sample t-test was used to analyse binary variables (AR or OT, cannula attempts) and one-way ANOVA used for data with multiple groups (preoxygenation, alarm activation and staff responses). To determine if any of the factors were predictive of anxiety, all five variables were each analysed using a multivariate general linear model technique with the remaining variables as covariates. Significance was assumed for p values <0.05. Qualitative responses were summarised systematically into worries, concerns and factors ameliorating/worsening anxiety. Thematic analysis, as described by Braun and Clarke (2006), was used to analyse the transcripts from the ten interviews. First, all transcripts were read then, beginning with the first interview, content of interviews was coded into major themes and sub-themes. Themes were constantly refined and re-ordered during the process of analysis. Thematic analysis of the postoperative interviews generated 14 codes (supplemental material 1), classified and described in the ‘Findings’ section under five meta-themes: anticipating the operation, preassessment, preparation for theatre in ward or admissions lounge, the experience of awaiting the procedure and addressing anxiety immediately prior to going to sleep. They are described in more detail in the ‘Findings’ section and supplemental material.

## Results

Forty-six patients were approached to participate. Two declined study participation, one patient was withdrawn because no blood pressure reading was taken prior to induction, two were withdrawn because the operative procedure was changed after study consent rendering the patient ineligible and one was withdrawn due to postoperative delirium.

Of the 40 participants, 19 (48%) were female, four (10%) had had no previous GA, and 42.5% were <40 years, 45.0% were 40–69 years and 12.5% were >70 years of age. One patient arrived in theatre with a cannula in place so only 39 patients were analysed for cannula attempts and in 15 patients there were no alarms in the AR and so staff responses were not recorded. Results of the observations made during induction of anaesthesia are shown in Table 1 and the measures of anxiety in Table 2.

**Table 1:** Pre-induction interventions observed and mean (SD) results for anxiety measures

Intervention					
Place of induction	OT	AR			
n	12	28			
ΔRPP (%)	28.4 (24.6)	39.3 (29.8)			
STAI	34.4 (12.3)	35.6 (13.0)			
Preoxygenation	None	LFM	TFM		
n	1	20	19		
ΔRPP (%)	22.3 (–)	34.5 (16.4)	38.4 (38.2)		
STAI	40.0 (–)	35.0 (14.4)	35.3 (11.1)		
Alarm noise	0	1	2	3	4
n	15	15	8	1	1
ΔRPP (%)	36.0 (21.1)	33.4 (35.0)	39.9 (33.6)	35.1 (–)	48.1 (–)
STAI	36.2 (12.3)	38.2 (14.2)	31.3 (9.4)	20.0 (–)	23.2 (–)
Staff responses	0	1	2		
n	7	8	10		
ΔRPP (%)	25.0 (21.0)	42.0 (31.0)	39.1 (40.6)		
STAI	30.0 (12.6)	34.2 (14.3)	38.3 (12.4)		
Cannula attempts	1	> 1			
n	33	6			
ΔRPP (%)	32.5 (25.2)	54.9 (41.8)			
STAI	36.6 (13.10	30.6 (7.1)			

AR: anaesthetic room; LFM: loose-fitting facemask; OT: operating theatre; STAI: state-trait anxiety inventory; TFM: tight-fitting facemask; ΔRPP: change in rate-pressure product.

**Table 2:** Comparison of anxiety measures recorded

Measure	Min	Max	Mean	SD
STAI	20	63	35	12.6
VASA (mm)	0	96	24	20.7
ΔRPP (%)	−18	121	36	28.5

STAI: state-trait anxiety inventory; VASA: visual analogue scale; ΔRPP: change in rate-pressure product.

The STAI and VASA scores were correlated (r = .786, P < 0.00) so only STAI scores were used for further analysis. Change in RPP did not significantly correlate with either STAI (r = −.145, p = .37) or VASA (r = −.105, p = .52). None of the pre-induction interventions were significantly predictive of stress levels (Table 3) with the largest effect seen being that of preoxygenation method on RPP with an r^2^ value of 0.12 (p = 0.331). To identify if there were any particular groups of patients susceptible to anxiety due to their surroundings, subjects were retrospectively divided into three groups using their STAI scores into low (≤30), normal (>30–49) or high (≥50) scores. The characteristics of these groups are shown in Table 4.

**Table 3:** Results of general linear model for predictive value of pre-induction interventions on two measures of anxiety

Intervention	ΔRPP		STAI		
	r squared	p	r squared	p
OT or AR	.086	.520	.061	.688
Cannula attempts	.070	.623	.062	.678
Preoxygenation	.120	.331	.059	.704
Alarm activation	.088	.508	.034	.871
Staff response	.079	.565	.062	.678

AR: anaesthetic room; OT: operating theatre; STAI: state-trait anxiety inventory; ΔRPP: change in rate-pressure product.

**Table 4:** Patient characteristics and pre-induction interventions according to anxiety levels

	Low anxiety	Normal anxiety	High anxiety
Male/Female	10/8	8/8	3/3
Previous GA/no previous GA	16/2	14/2	6/0
Age (yr) <40/40–69/>69	7/9/2	8/6/2	2/3/1
OT/AR induction	13/5	11/5	4/2
Cannula attempts 1/>1	14/3	13/3	6/0
Preoxygenation LFM/TFM	11/7	5/10	4/2
Alarm activations 0/1/>1	6/6/6	7/5/4	2/4/0
Staff response 0/1/2	5/4/3	1/3/5	1/1/2

AR: anaesthetic room; GA: general anaesthetic; LFM: loose-fitting facemask; OT: operating theatre; TFM: tight-fitting facemask.

We found a lack of correlation between patient-reported anxiety and sympathetic activity indirectly measured by RPP. This may suggest that by the time patients were awake enough to complete the questionnaire in PACU their response was not a true reflection of their stress at the time of induction – once the anaesthetic and operation are successfully completed the relief being experienced by the patient may have significantly influenced how they reported feeling before going to sleep. However, a lack of correlation between physical measures of stress and patient rating scales is well known from studies of patients with generalised anxiety disorder, such as McLeod et al (1986), suggesting that our results from PACU may be reflective of patient anxiety at induction. These results also suggest that the ideal measure of stress in the perioperative period is unknown. Given this disconnect between anxiety and physiological stress, future studies of patient experiences should consider using both, as both have implications for perioperative care. Perceived anxiety negatively impacts on a patient’s experience of the anaesthetic and operation, including being associated with increased postoperative pain. Physiological stress associated with surgery increases perioperative cardiovascular complications, with a variety of techniques having been tested to attenuate this effect.

## Findings

The qualitative data also provided little suggestion that pre-induction interventions were contributing to anxiety. From the PACU questionnaire, one patient reported ‘hearing heart rate going up, feeling panicky’ as their last thought before sleep; two patients were still distracted by pain from the cannula insertion at induction; three patients commented about having the mask over their face, with one describing this as ‘weird’ and another felt the mask ‘stopped them replying’ to the questions being asked by the anaesthetic team. In the structured interviews, the oxygen mask was mentioned by one patient and the cannula insertion by six patients, but neither were described as a source of anxiety. The alarm noises and staff responses were not mentioned.

Participants varied in terms of their reported anxiety concerning the operation and their preferred approach to coping. Some had clear memories of the preoperative information given to them and were able to articulate how helpful that had been. Others had no clear memories of the information and confided that they preferred to ‘get on with it’. No participants had any negative comment about any member of the team and there was a general appreciation of the efforts made to make the patient fully informed before the operation and comfortable physically and mentally prior to induction. The data are summarised in detail under the following five themes: anticipating the operation, preassessment, preparation for theatre in ward or admissions lounge, the experience of awaiting the procedure and addressing anxiety immediately prior to going to sleep. They are described in more detail in the supplemental material.

1. Anticipating the operation. All participants discussed their feelings before the operation. Eight had had previous surgery and three of these thought that this made them more anxious when they had had a prior bad experience. Two others thought that it helped, giving them a sense of what to expect. ‘the first time I think it was very difficult knowing you’d go in there and be put to sleep and would you wake up again and all’ (Participant 2).

Six mentioned having particular fears about the surgery, though these tended to be focussed on the anaesthetic rather than the surgery itself. Three felt generally anxious and three felt worried about waking up after the operation: ‘How are they going to bring me out of this? Can they bring me out of this, being so deep?’ (Participant 4).

Two participants worried about waking up *during* the operation, and one had previously experienced this.

For two people, the anxiety was particularly powerful and focussed on loss – on a fear of dying and leaving family behind: ‘it’s leaving people behind, you know?’ (Participant 3).

For one of these participants, this anxiety was higher than they had experienced when having surgery before. They felt this was because they now felt closer to their family, particularly young children, and this was tied up with feeling older: ‘the older you get, the more concerned you get’ (Participant 1).

2. Preassessment practices. Seven participants explicitly mentioned how good the preassessment staff were at making them feel at ease but there were mixed views about how helpful it had been. Five felt it had been helpful and many remembered asking several questions. Two felt it had been good for learning about the practicalities on the day of the operation but less helpful for anxiety, though one participant felt that they were given less practical information because they were a nurse and people assumed that they knew it already. One participant acknowledged the importance of accurate information: ‘I’m a dr-googler which is a bit wrong like’ (Participant 5).

Two people found the information booklet helpful and one reported that it had calmed their fears, but two felt they knew the information already and six had no clear recollection: ‘I think I looked at them briefly’ (Participant 6).

Several were unclear about how useful the written information was to them: ‘Some were, some weren’t really’ (Participant 9).

3. Preparation for theatre in ward or admissions lounge. Participants were also asked about visits from the anaesthetist or surgeon prior to surgery. Eight said that this had been helpful, though one could not recall who they had spoken to: ‘he put my mind at ease’ (Participant 8).

Several participants talked about being able to ask any questions that they had. One was reassured by knowing that the anaesthetist and surgeon knew each other and worked closely together. For those that had acknowledged feeling anxious, two felt these conversations particularly helpful: ‘Because it was explained to me I knew what was going on, so I wasn’t really nervous’ (Participant 7).

Three participants saw these conversations explicitly as a useful way of helping them actively prepare for the operation: ‘you start to plan it’ (Participant 5).

4. The experience of awaiting the procedure. Participants talked about their experience of the operation, with several naming the wait before going to the theatre as the most difficult aspect: ‘I’d gone well over 24 hours and I wanted a drink’ (Participant 3).

Participants reported frustration at not knowing how long they were going to wait, and one talked about considering going home. When actually in theatre, three participants reported no feelings of anxiety but six others did, including two who felt panic: ‘I felt a bit panicky because it was the time then that I couldn’t turn back’ (Participant 3).

For one, however, the anxiety was manageable and understandable: ‘I think it’s natural to have a base-level of concern’ (Participant 10).

5. Addressing anxiety immediately prior to going to sleep. The fifth theme relates to the thoughts of the participants on the way the team treated them during preparation for induction, in particular their attempts to contain any anxiety.

Almost all of the participants mentioned how important it was that they were introduced to the team: ‘personally, it makes it easier – you put a face to somebody’ (Participant 5).

They also found team members approachable and responsive, and non-judgemental when they talked about their anxiety: ‘and I told her what my fears were but she never judged me or anything, she were lovely’ (Participant 3).

There were mixed views about whether the reassurance given by members of the team worked. Two felt reassured that the team were there throughout; two others recognised that they still felt anxious regardless of what was said.

There was particular discussion about the atmosphere in the AR, with seven participants commenting on the warmth and humour they felt, and how that helped them to relax: ‘it didn’t seem like a conveyer belt’ (Participant 5).

Six participants discussed how the team had helped them with their anxiety, with two recognising that they distracted them from their worries by talking to them: ‘they talk about you, your personal life, other stuff…your focus just goes off it, to be honest with you, it’s not on the anaesthetic, it’s on other stuff so you’re kind of comfortable with it’ (Participant 2).

Four participants acknowledged the importance to them of being talked through the procedure as it happened: ‘I really like the way the consultant walked me through the sensations’ (Participant 10).

Overall anxiety levels in our patients were close to the normal range, with only six of 40 patients registering a clinically severe anxiety response (Bekker et al 2003). This is reassuring, suggesting patients are appropriately prepared for their anaesthetic by the combination of written and verbal information provided to patients at preassessment, followed by a preoperative consultation with their anaesthetist. Patients with high levels of anxiety (Table 4) appeared from the qualitative data to have differing sources of anxiety. Almost all the participants expressing a worry about waking up during or after the procedure or their family were clinically anxious (5/6), while almost all who were in the normal anxiety range were thinking about the procedure and/or engaged in a distraction activity (33/34).

## Discussion

### Limitations

Anxiety levels at the time of induction of anaesthesia are infrequently studied, and the challenges of doing so mean there are limitations to our findings. This was a single centre, pragmatic study with no attempt to control preoperative patient information, anaesthetic interventions or staff behaviour. Additionally, we did not recruit patients based on disease or operation they were to undergo. This was a deliberate strategy to allow us to quantify anxiety in the ‘normal’ patient population, but which of course limits the ability to extrapolate our results to other units where processes and behaviours may be quite different. All GAs observed were being directly supervised by experienced anaesthetists. Again, this was a deliberate plan to avoid the presence of a consultant anaesthetist investigator at the time of induction intimidating junior anaesthetists and so potentially changing their behaviour. Of course we have no way of knowing if our presence, or the simple fact that the patient was enrolled in the study, changed how the theatre staff treated their patients, but this is unavoidable without the ethically challenging strategy of covert observation.

We used the STAI/VASA measures retrospectively which is not how these tools were originally intended to be used; they normally ask about feelings at the moment when they are being completed, with the subject being encouraged not to think about their response in detail but answer based on their ‘gut reaction’. In the situation being studied here, ie immediately before unconsciousness, this was not possible. That was why we chose to also measure the cardiovascular response to stress, which reflects the physiological response to stress at the time. However, the qualitative responses provided postoperatively suggest validity between patient responses and their reported experience.

The reported sources of anxiety in our study are similar to those reported elsewhere, for example fear of either waking up during the procedure, not waking up at all or concerns for family (Caumo et al 2001). Although none of the interventions in the AR were significantly correlated with high levels of anxiety, the largest effect was the preoxygenation method in keeping with Mitchell (2008) who also found the preoxygenation facemask to be a source of anxiety. Although previous work has shown prior perioperative experience to be beneficial, our results were mixed, with the nature of the experience (good or bad) being relevant, although of note, none of the patients in our high anxiety group had undergone surgery before. In contrast to existing work (Erkilic et al 2017), we did not find a correlation between anxiety and either age or sex. Patient-reported anxiety levels in our study were comparable with other studies such as Oldman et al (2004) who measured anxiety on the morning of surgery (mean STAI ≈33). However, Ahmetovic-Djug et al (2017) investigating preoperative anxiety found a higher mean STAI (53). Only 15% of our study population achieved high anxiety scores on the STAI; this is lower than reported anxiety in other perioperative studies where the incidence of anxiety ranges from 60 to 92% (Perks et al 2009). This difference may represent the fact that our scores were measured in the recovery area postoperatively as described above, although work by Johnston (1980) does not support this as anxiety levels remained high for some days postoperatively. Many studies undertaken to investigate the causes of perioperative anxiety are disease-or operation-specific and it is difficult to extrapolate the results to a wider population (Stamenkovic et al 2018). This is also true of single centre studies or those carried out in different healthcare systems as the degree of anxiety is affected by institution-specific factors such as the preoperative arrangements and the information given.

Some comments were provided about what more could be done by the team to keep people calm. There were two types of issues raised, one around providing more information about procedures (eg waiting times, masks and beeping noises while going to sleep) and one about discomfort experienced during preparation. The uncertainty of knowing when the patient was actually going to leave the ward to go to theatre was a source of stress for some patients. This is clearly a challenging time for the patient; after the frenzy of preoperative visits and preparation after arriving on the ward, they are then left alone with minimal interaction with the staff, and with great uncertainty about when they will be going to theatre. Uncertainty in general, and in particularly not knowing when the current worry will end, are particularly strong provokers of anxiety. We believe there is significant potential to improve the information given to patients to alleviate this situation.

The qualitative data suggested all patients felt involved in their care, and there was a consistent message about factors that reduced anxiety such as the friendly atmosphere generated by the team, chatting with patients, providing explanations for what was happening and receiving information that informed them about the different stages of the procedure. There was agreement that being introduced to the team was helpful but experiencing some degree of anxiety in response to a stressful event is normal human behaviour, and there was some indication that not all preoperative anxiety will be alleviated by good communication practices (Bekker et al 2003). A degree of anxiety may even be considered positive; some increase in arousal is associated with better recall and systematic evaluation of information whereas very low or high levels are related to less optimum processing strategies, ie the inverted U-shaped relationship between arousal and information processing ability (Bekker et al 2003). Establishing an individual patient–anaesthetist relationship preoperatively seems to have been helpful in facilitating a personalised approach to reducing anxiety prior to induction as described below. Late changes in allocation of anaesthetist to lists to cover sick leave, etc, which are common in our department, would break this relationship and so potentially worsen the patient’s experience. The behaviour of the anaesthetic team immediately before induction seems to have worked well with patients commenting on being distracted from the impending induction by the conversation and most being reassured by the team. The observers witnessed a diverse range of strategies being used by anaesthetists to mitigate anxiety during induction. In many cases, this involved significant personalisation of care, ie modifying the technique according to individual patient’s responses to the standard ‘patter’. The reassurance provided before induction of anaesthesia was not just based on the verbal narrative used, but also ascribed to the general atmosphere and the ‘the feel’ of the team. This demonstrates that most of the staff had good rapport and communication with the patients.

Based on both quantitative and qualitative results the anaesthetic clinical interventions performed immediately before induction had little impact on patient anxiety or stress. It is reassuring that these now ubiquitous safety measures appear in our study not to have a detrimental effect on the patient experience. Only two patients commented on alarm noises despite these occurring in 25/40 inductions. Despite this, staff responses to the alarms were varied and could be improved, with less than one third resulting in specific reassurance being given to the patients that the alarms were not a cause for concern.

## Conclusions

Practical interventions performed on patients during induction of an anaesthetic do not cause anxiety and most patients seem unaware of them. Anxiety levels at anaesthetic induction are similar, and possibly lower, than earlier in the preoperative period, possibly because by this stage patients have little influence over their situation and are being actively reassured and distracted by a whole team. Our results are reassuring, suggesting that theatre staff are good at alleviating anxiety at this potentially stressful time. However, our study also suggests that more could be done to avoid preoperative sources of anxiety such as better planning in the immediate preoperative period, eg to avoid uncertainty for the patients about the timings of their operation.

## Supplemental Material

sj-pdf-1-ppj-10.1177_1750458920936933 - Supplemental material for Investigating the causes of patient anxiety at induction of anaesthesia: A mixed methods study Click here for additional data file. Supplemental material, sj-pdf-1-ppj-10.1177_1750458920936933 for Investigating the causes of patient anxiety at induction of anaesthesia: A mixed methods study by Andrew B Lumb, Gary J Latchford, Hilary L Bekker, Anna R Hetmanski, Caroline R Thomas and Claire E Schofield in Journal of Perioperative Practice

## Notes

*No competing interests declared*

## Supplemental data

Supplemental data related to this article consists of a file showing details of the thematic analysis of the structured interview transcripts and the three Appendices.
