# Supplementary material for: Investigating the causes of patient anxiety at induction of anaesthesia: A mixed methods study
Source: J Perioper Pract. 2020 Jul 8;31(7-8):246–54. doi: 10.1177/1750458920936933 (PMC8258711; doi:10.1177/1750458920936933)
Supplement: sj-pdf-1-ppj-10.1177_1750458920936933 - Supplemental material for Investigating the causes of patient anxiety at induction of anaesthesia: A mixed methods study [file sj-pdf-1-ppj-10.1177_1750458920936933.pdf]

Supplementary material. Codes developed from thematic analysis of the qualitative data obtained from the postoperative interviews.

**Anticipating the operation**		
Previous experience<br><br>Yes 1 2 3 5 6 7 8 10<br>No 4 9	Helps – 2 3<br>Doesn't make a difference – 5 4 8 10<br>Worse 1 6 7	• "recovery a bit longer than I expected this time" 2<br>• "I've done it before, it didn't come as a surprise to me. But the first time I think it was very difficult knowing you'd go in there and be put to sleep and would you wake up again and all" 2<br>• "A lot of concerns, because that (previous op) wasn't a very good experience at all." 6
Importance of family and fear of loss	1 3	• "I'm more concerned about family and waking up, not waking up" 1<br>• "I've got young grandchildren and I was scared in case what happened" 3<br>• "it's leaving people behind, you know?" 3<br>• Q: it was a reminder of things? A:"yes, of what you can lose" 3
Anxiety	Yes 1 3 10<br>No 4 5	• "the older you get, the more concerned you get" 1<br>• "Waking up and what's going to happen" 1<br>• "worried ..with my neck" 4<br>• But otherwise "I wasn't anxious about nothing" 4<br>• "No, because I'd been put at ease by the steps leading up to it" 5<br>• 10: worried would be confused by anaesthetic when came round
	About coming round 1 3 4	• "It was coming round from the anaesthetic" 3<br>• "How are they going to bring me out of this? Can they bring me out of this, being so deep?" 4<br>• "not waking up" 1
	About waking up during the operation 2 6	• 6 had previously had this experience
**Pre-assessment**		
The staff were lovely	1 2 3 5 6 7 10	• "she made me feel really

		comfortable" 2<br>• "it were fine" 5<br>• "friendly and good" 6<br>• "pretty straight forward" 7
Helpful?	good for practicalities, not for anxiety 3 6<br>needed more on practicalities 9 (who was a nurse)<br>Yes, asked lots of questions 5<br>Can't remember 4<br>"Definitely made it a lot easier" 2<br>Eased concerns 7<br>No 6 9<br>no fears at all 8<br>really helpful 10	• "I'm a Dr-googler which is a bit wrong like" 5<br>• "I think it makes you feel a bit more at ease if you can ask questions and stuff" 5<br>• "goes in one ear and out the other" 4<br>• "They wouldn't have been in a position to explain processes to me" 6<br>• "it was a bit rushed and that made me nervous" 9
	Discussed anxiety<br>3 7	
	Bringing family member is helpful<br>3	
The Information booklet		
	Knew the information already<br>1 3	• "Not very helpful" 1
	informative<br>5 7	• "Helped prepare for the operation" 5
	Helped calm my fear<br>3	
	Not sure<br>2 6 8 9 10	• "I think I was given a booklet" 2<br>• "I think I looked at them briefly" 6<br>• didn't think they had been given anything 8<br>• "some were, some weren't really" 9<br>• "if I'm honest with you I didn't hang too long on that side of things, because I thought, well it is what it is, kind of thing" 10
	Didn't retain information<br>4	
**Preparation for theatre in ward or admissions lounge**		
Talking to the anaesthetist/surgeon before the operation	Helpful to talk to:<br>Anaesthetist/surgeon:<br>1 5 7 8 9 6 10<br>'manager': 2	• "any questions I had she answered" 2<br>• "he listened again to my concerns again, he tried to put my mind to ease" 6

		• "seeing them together, my surgeon and anaesthetist, and realising that they had previously known each other, worked together and that there was a professional relationship and a good one, you could see that. I don't know if that's something they put out for patients but it gave me confidence" 6<br>• "Because it was explained to me I knew what was going on, so I wasn't really nervous" 7<br>• "he put my mind at ease" 8<br>• "I always ask questions" 10<br>• "I don't remember a conversation about the recovery room specifically" 10<br>• "I don't think I had any issue" 7<br>• "it was all quite straightforward" 8
Preparation is helpful	2 5 6	• "I think I was prepared for the anaesthesia but not for the waking up" 2<br>• "you start to plan it" 5<br>• "I think it really is helpful" 6<br>• Participant 9 is a nurse and wondered whether people may have assumed knowledge
**The experience of awaiting the procedure**		
Waiting was the biggest thing	3 5 4<br><br>6 got in early and was grateful was in a bed, rather than at home<br><br>5 smoked to relax<br><br>9 confirmed it was difficult, even though their wait was short	• Physical discomfort when waiting – especially thirst<br>• "I'd gone well over 24 hours and I wanted a drink" 3<br>• "I nearly got dressed and went home" 3<br>• Told different things 4<br>• "more frustration than being anxious" 5<br>• "It just makes you feel really nervous; you know it's coming but you don't know when. That's what's not nice" 9<br>• "It's getting told different little things sometimes" 3 (about the duration of the wait)
Anxiety	Loss of control and panic<br>3	• "I felt a bit panicky because it was the time then that I couldn't..turn back" 3<br>• Dizziness sparked panic 3

	'nervous'<br>2<br>Anxiety just before the operation<br>1 6 7 10	• "I think I got a nervous at the end." 2<br>• "in that one room you're looking round and thinking 'oh God' and you start panicking a bit" 1<br>• "when I started being stressed when someone came to pick me up from the bed, and I was on the bed in the pre-op room in about thirty seconds and then everything happened very very quickly" 6<br>• "Are you going to come back alive" 7<br>• "I think it's natural to have a base-level of concern" 10
	No anxiety<br>5 4 8	• "I'm not a very worrying person" 5<br>• "a little bit" 4
**Addressing anxiety immediately prior to going to sleep.**		
Talking to the team	Being introduced is good 1 2 5 4 6 7 9 10	• "personally, it makes it easier -you put a face to somebody" 5<br>• "its nice to see someone you know" 4<br>• "I know it's all silly because it's only a couple of minutes of meeting someone but it is good." 1<br>•
	Very approachable (e.g. anaesthetist)<br>1 3	• "People, how they talk to you. And get on with you. It's like they know you straight away. I mean, don't get me wrong, they do this day in and day out, but they know what to say to people." 1<br>• "it was comfortable from beginning to end" 2<br>• "Good atmosphere, all round to be honest with you…top to bottom" 2
	Non-judgemental<br>3	• "and I told her what my fears were but she never judged me or anything, she were lovely" 3
	Responsive<br>3	•
Reassurance	doesn't work<br>1 3	• "it's something probably that you go through " 1<br>• "I still felt anxious anyway, you know" 3<br>• "Well I don't listen to nobody anyway" 3<br>• "up to that stage I was all panicking, and I thought 'just get on with it'" 1
	does work<br>2 6	• Reassured because told ""we will be present within the room"2<br>• Helped talking to surgeon 6

Atmosphere in anaesthetic room	Humorous, relaxed, nice people<br>1 3 5 7 8 9 10	• "it wasn't solemn!" 5<br>• "seeing them relaxed (the staff) makes you feel more relaxed" 5<br>• "it didn't seem like a conveyer belt" 5
	Distracted me from worries<br>2 7	• "they talk about you, your personal life, other stuff…your focus just goes off it, to be honest with you, it's not on the anaesthetic, it's on other stuff so you're kind of comfortable with it." 2 (distraction)<br>• "it takes your mind off it" 7
	Helpful<br>1 3 4 6	• "when I went through into that room they talked to me and everything and it were fine, it was good. " 1
Being talked through procedure	2 6 9 10	• "as they were going along they talked through the process, everything as they were doing it." 2<br>• "they talk you through it as they're going along" 2<br>• "I really like the way the consultant walked me through the sensations" 10

Appendix 1 - Clinical observation checklist.

1. The patient’s heart rate and blood pressure in the preassessment clinic and maximum values achieved while awake prior to induction

2. Whether the patient was induced in the anaesthetic room (AR) or theatre (OT)

3. Preoxygenation: classified as none, via a loose face mask (LFM) or a tight-fitting face mask (TFM)

4. Alarm activations i.e. the number and volume of equipment alarms sounding classified into

   0 - no alarm noise throughout

   1 - occasional background/quiet alarm

   2 - occasional loud alarm

   3 - intermittent but repeated and intrusive alarms

   4 - constant loud alarms, no significant quiet periods.

5. Staff response (interactions with the patient in response to any audible alarms):

   0 - alarms silenced and the patient reassured

   1 - alarms silenced but not commented upon to patient

   2 - alarms ignored by all staff

6. The number of attempts at cannulation, recorded as either one or more-than-one attempt.

Appendix 2. Patient reported experience of preoperative anxiety questionnaire

Please mark this line to indicate how anxious you felt as you went to sleep:

Zero anxiety Maximal anxiety

Please answer the following questions: Immediately before you went to sleep:

1. what were you thinking about?

2. did you feel involved in your care or did you feel ignored?

3. was there anything that made you feel more tense or stressed?

4. was there anything that made you feel more calm or relaxed?

5. was there anything we could have done to help you feel more calm or relaxed?

Appendix 3. Patient reported experience of preoperative anxiety interview schedule.

1. Was this your first anaesthetic?
   a. If yes, go to question 2
   b. If no, did you have any particular concerns based on your previous experience(s)?
2. To what extent were any concerns about having an anaesthetic eased or made worse by:
   a. Your preassessment visit
   b. Any leaflets or other written information provided preoperatively
   c. Your preoperative visit by your anaesthetist?
3. Did you feel tense or stressed while being put to sleep? If no, go to question 4.
   a. Were your main concerns about the anaesthetic, and if so what worried you about it?
   b. If the anaesthetic didn't worry you, did something else cause you to be tense or stressed as you went to sleep?
4. How would you describe the atmosphere in the anaesthetic room as you were being put to sleep?
5. Is there anything we could have done differently to improve your experience?
